# An oral health literacy intervention for Indigenous adults in a rural setting in Australia

**DOI:** 10.1186/1471-2458-12-461

**Published:** 2012-06-20

**Authors:** Eleanor J Parker, Gary Misan, Alwin Chong, Helen Mills, Kaye Roberts-Thomson, Alice M Horowitz, Lisa M Jamieson

**Affiliations:** 1 Australian Research Centre for Population Oral Health, School of Dentistry, University of Adelaide, Adelaide, Australia, 5005; 2 University of South Australia, Whyalla campus, Whyalla, Australia; 3 Menzies School of Health Research, Charles Darwin University, Darwin, Australia; 4 School of Public Health, University of Maryland, College Park, MD, USA

## Abstract

**Background:**

Indigenous Australians suffer substantially poorer oral health than their non-Indigenous counterparts and new approaches are needed to address these disparities. Previous work in Port Augusta, South Australia, a regional town with a large Indigenous community, revealed associations between low oral health literacy scores and self-reported oral health outcomes. This study aims to determine if implementation of a functional, context-specific oral health literacy intervention improves oral health literacy-related outcomes measured by use of dental services, and assessment of oral health knowledge, oral health self-care and oral health- related self-efficacy.

**Methods/design:**

This is a randomised controlled trial (RCT) that utilises a delayed intervention design. Participants are Indigenous adults, aged 18 years and older, who plan to reside in Port Augusta or a nearby community for the next two years. The intervention group will receive the intervention from the outset of the study while the control group will be offered the intervention 12 months following their enrolment in the study. The intervention consists of a series of five culturally sensitive, oral health education workshops delivered over a 12 month period by Indigenous project officers. Workshops consist of presentations, hands-on activities, interactive displays, group discussions and role plays. The themes addressed in the workshops are underpinned by oral health literacy concepts, and incorporate oral health-related self-efficacy, oral health-related fatalism, oral health knowledge, access to dental care and rights and entitlements as a patient. Data will be collected through a self-report questionnaire at baseline, at 12 months and at 24 months. The primary outcome measure is oral health literacy. Secondary outcome measures include oral health knowledge, oral health self-care, use of dental services, oral health-related self-efficacy and oral health-related fatalism.

**Discussion:**

This study uses a functional, context-specific oral health literacy intervention to improve oral health literacy-related outcomes amongst rural-dwelling Indigenous adults. Outcomes of this study will have implications for policy and planning by providing evidence for the effectiveness of such interventions as well as provide a model for working with Indigenous communities.

## Background

Indigenous Australians include people who identify as being of Aboriginal or Torres Strait Islander descent, representing 2.5% of the total Australian population in 2006 [1]. They are a diverse population, belonging to many distinct language groups and living in a wide variety of locations [2]. The majority of Indigenous Australians live outside major cities, with 43% living in regional and 25% in remote areas.

Indigenous Australians suffer from poorer oral health than non-Indigenous Australians. National estimates indicate that Indigenous Australian adults have higher rates of total tooth loss, higher percentage of reported toothache, lower mean number of dental visits, are more likely to visit for a problem rather than for a check-up and receive a lower mean number of dental fillings compared to non-Indigenous Australians [3]. Indigenous children experience, on average, twice the level of dental caries in both the deciduous and permanent dentitions with more untreated decay than their non-Indigenous counterparts [4]. In addition, at all ages between 4 and 15 years, a greater percentage have experienced dental caries when compared with their non-Indigenous counterparts [2]. Non-metropolitan Indigenous children and the more socially disadvantaged are even more severely positioned in terms of oral health outcomes [5-7].

### Previous work

Previous oral health research with Indigenous adults in Port Augusta has revealed important findings [8-10]. Initial qualitative investigations identified a strong sense of powerlessness, with participants feeling a lack of control over their oral health and health care decisions, at both the individual and community level [8]. There was a clear perception that behaviours promoting oral health were not widely practised and that significant barriers to dental care existed together with fatalistic views about oral health [8]. In the later study, a convenience sample of 468 participants completed a self-report questionnaire, including the REALD-30 to measure oral health literacy [10]. This study revealed associations between oral health literacy and self-reported oral health. Lower oral health literacy scores were associated with poor oral health literacy-related outcomes, including a belief that either that teeth didn’t need to be brushed or only needed to be brushed once a day; that cordial (flavoured sugary drink) was good for teeth; and that people didn’t have their own toothbrush, or that even if they owned a toothbrush had not brushed the previous day. Each of these oral health literacy-related outcomes was in turn associated with poor self-reported oral health. In addition to the research findings, this study demonstrated that conducting oral health research utilising self-report questionnaires was successful in this community.

Oral health literacy, like general health literacy, incorporates the capacity a person has to learn and use information about oral health in making decisions about their oral health. Developing adequate levels of health literacy may depend on external factors such as education, experiences in health settings and family attitudes; and individual factors such as cognitive ability and prior knowledge [11]. Lower levels of health literacy are commonly found in people who have low levels of education and income or have a different first language [12]. These characteristics are prevalent in the Australian Indigenous population.

Having poor oral health literacy can bring significant challenges. A recent study in the United States described how caregivers with low oral health literacy displayed low levels of oral health knowledge and poor self-reported oral health, which was reflected in their children who also had sub-optimal oral health with related poor oral care behaviours [13].

Targeted interventions that used clear communication and tailored and supportive training techniques have had some success in improving health outcomes for people with low health literacy. One such intervention with diabetic participants reported enhanced and retained management skills with improved glycaemic control [14]. To date, there have not been any studies which involve interventions targeting oral health literacy in Indigenous populations.

Like health literacy, a number of screening tools have been developed to determine levels of oral health literacy. Some health literacy tools have been criticised for being too narrow in their range of testing, or relying heavily on the participant’s ability to read [15]. This might also apply to their equivalents in dentistry. The Rapid Estimate of Adult Literacy in Dentistry (REALD-30) is a 30 item questionnaire that screens the participant’s ability to read dental terminology with correct pronunciation [16]. The Test of Functional Health Literacy in Dentistry (TOFHLID) tests reading comprehension and numeracy skills [17]. These instruments have limited use in individuals who demonstrate low literacy and numeracy skills or where English is not their first language. These characteristics are not uncommon in older Indigenous Australians.

Given the limitations of health literacy screening tools, a new tool has recently been developed in Australia. The Health Literacy Measurement Scale (HeLMS) takes a broad approach to measuring health literacy, addressing many of the limitations of other health literacy tools [18]. The HeLMS was developed using a health literacy conceptual framework developed from a patient perspective. Consisting of 29 items, each rated on a 5 point Likert scale, the HeLMS scores 8 domains: patient attitudes towards their health, understanding health information, social support, socio-economic considerations, accessing general practitioner health care services, communicating with health care professionals, being proactive and using health information [18]. Using the HeLMS, people with chronic lower back pain were found to have lower scores for the domain assessing patient attitudes towards their health than those without chronic back pain, as well as lower scores for each item within that domain [18].

### Aims

This study assesses oral health literacy and self-reported oral health outcomes among rural-dwelling Indigenous adults and will determine if implementation of a functional, context-specific oral health literacy intervention improves oral health literacy-related outcomes. For the purposes of this study, oral health literacy-related outcomes include use of dental services, oral health knowledge, oral health self-care and oral health-related self-efficacy.

Specifically, the aims are to:

· Describe the extent of poor oral health literacy among rural dwelling Indigenous adults

· Describe the relationship between oral health literacy and oral health literacy-related outcomes

· Determine if a functional, context-specific oral health literacy intervention improves oral health literacy

· Determine if a functional, context-specific oral health literacy intervention improves oral health literacy-related outcomes

## Methods/design

### Study design

**Figure 1:**
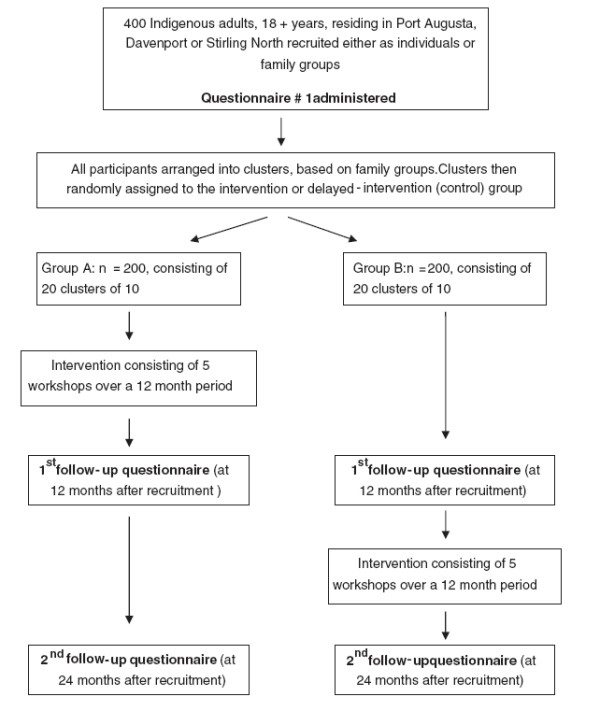
Study design schema.

This study is a randomised controlled trial, utilising a delayed intervention design where all participants will ultimately be offered the intervention. A study schema is presented in Figure 1.

### Setting and location

The study is situated in Port Augusta, South Australia, and includes participants from outlying communities who frequent services in Port Augusta.

### Participants

To be eligible participants must be Indigenous, aged 18 years and above, and intending to reside in Port Augusta or a nearby community for the duration of the study (two years).

### Recruitment

Participants are recruited using a variety of methods previously used successfully with this community [10] including self-nomination, home visits, word of mouth, visits to community centres and referrals. Promotion of the study has occurred via posters in community centres and advertisements on the local Aboriginal radio station.

### Funding

Funding has been provided by the National Health and Medical Research Council of Australia (NHMRC, project grant 627101).

### Staff

A total of four part-time staff are employed for the initial phase of the study including a dental therapist, employed as a project manager. The remaining three staff are of Indigenous descent and are employed as project officers. In addition to recruiting participants, administering questionnaires and delivering the intervention, the Indigenous research officers play an invaluable role in providing ongoing cultural advice for investigators. The Indigenous project officers have been provided with basic oral health theory training by the project manager, enabling them to deliver the intervention. All staff have lived or worked in the local area previously.

### Advisory group

An advisory group has been established comprising seven Indigenous community representatives. The group includes people working in health and education as well as community Elders. The advisory group has provided input into the study design, promotion of the study and the data collection instruments and techniques. The advisory group continues to advise investigators and the study team in relation to appropriate implementation of the study in the local community.

### Pilot study

A pilot study was conducted in a neighbouring regional centre, primarily for the purpose of field testing the intervention instruments and giving the Indigenous project officers experience in delivering the sessions. Participants were invited to be involved in the pilot study through contacts at the local Indigenous health service. Written consent was obtained and participants received supermarket vouchers in acknowledgement of their time commitment. Involvement included completion of a baseline questionnaire, attendance at group intervention sessions and completion of a follow-up questionnaire. Participants were asked for immediate feedback upon completion of the questionnaire and after each intervention session as well as at the completion of the pilot study. This feedback was used to refine the questionnaire and intervention instruments for the parent study.

### Consent and incentives

Participants are provided with written and verbal information about the study prior to giving consent. In acknowledgement of their time commitment, participants receive a $20 supermarket gift voucher upon completion of each questionnaire and a $10 gift voucher for each intervention session attended. At each intervention session refreshments are offered and participants provided with a variety of products to reinforce the key messages from each session, for example, water bottles, tooth brush and tooth paste, disposable dental mirrors.

### Intervention

The intervention consists of a series of five workshops delivered over a 12-month period.The instruments were developed collaboratively by the project manager, other study investigators and the Indigenous project officers, and are used to guide the project officers in delivering the intervention. Each workshop lasts approximately one and a half hours, including morning or afternoon tea and is conducted predominantly by the Indigenous project officers. Workshops consist of presentations, hands-on activities and interactive displays, group discussions and role plays. A key focus of the workshop series is on breaking down barriers and improving confidence of participants. Information around dental disease processes (ie dental caries, periodontal disease and dental erosion) is fundamental to workshop activities and discussion. The themes addressed in the workshops are underpinned by oral health literacy concepts and incorporate oral health-related self-efficacy, oral health-related fatalism, oral health knowledge, access to dental care and rights and entitlements as a patient.

### Randomisation

The cluster randomisation method was selected as an appropriate approach for this study because of its acceptability to the participants and the local community (based on feedback from the advisory group and from the pilot study), as well as for the potential to increase the efficacy of the intervention through encouraging discussion amongst family groups and providing a supportive environment for participants’ development or change.

After purposive recruitment of 400 individual participants largely through local knowledge of kinship and other networks of the Indigenous project officers, 40 groups are formed based on family and social groups. Group sizes range from 8–12 people. The Indigenous project officers are responsible for assigning participants to the groups, utilising their knowledge of the local community. Family groups (clusters) are randomly assigned on a 1:1 basis to either a test-immediate or control-delayed intervention group. A computer-generated permuted block randomisation sequence is used, developed by biostatisticians at the Australian Research Centre for Population Oral Health (ARCPOH) using a random number generator. Randomly selected block sizes of 4, 6 and 8 are used, such that there is an equal number of participants in each intervention arm within the blocks. This ensures that if the study is stopped at any particular time there will be approximately an equal number of participants in each intervention arm (Figure 1).

### Data collection

Data is collected through self-report questionnaires at baseline, at 12 months and at 24 months. The questionnaires include items pertaining to the primary and secondary outcomes and covariates. Questionnaires are administered by the Indigenous project officers and completed either as an interview or self-completed, with the degree of self-completion determined by the participant. The project officers are provided with a scripted method of introducing and administering the questionnaire. A log of attendance at the intervention sessions is collected.

#### Primary and secondary outcomes

The primary outcome measure is oral health literacy, measured using the HeLM [18] adapted by investigators for a dental context.

Secondary outcome measures include oral health knowledge, oral health self-care, dental service utilisation, oral health-related self-efficacy and oral health-related fatalism [19].

In the initial study design and pilot study, REALD-30 was utilised as the instrument to measure oral health literacy. After feedback from pilot study participants and the advisory group, it was deemed more culturally-appropriate to utilise the HeLM, adapted by study investigators and project officers for oral health, to measure oral health literacy. Specific feedback in relation to the use of REALD-30 included: (1) participants felt they were being tested and were hence intimidated; (2) the relevance of REALD-30 to the Indigenous oral health context was not clear; (3) the use of REALD-30 may be a barrier to full participation and completion of other components of the questionnaire.

#### Covariates

Socio-demographic covariates include age, gender, education level, employment, income source, number of people staying in the house the previous night, number of children under 18 living in the household and car ownership.

General health covariates include medical conditions, behaviours such as cigarette smoking and alcohol consumption status, and self-rated general health.

Oral health covariates include self-reported oral health, previous dental extractions and oral health-related quality of life.

Psychosocial covariates linked with oral health outcomes in the Australian population [20], include personal control [21] perceived stress [22] and an adapted version of the social support measure [23].

### Data handling and statistical methods

There will be three main analyses. The first analyses will occur after baseline, to quantify the extent of poor oral health literacy and ascertain the relationship between oral health literacy and oral health literacy-related outcomes. The second analyses will occur after 12 months, providing a comparison between the intervention and delayed intervention (control) groups, in order to assess the impact of the intervention. The third analyses will occur after 24 months, assessing the sustainability of intervention impact 12 months post-completion in the initial intervention group.

The Generalised Linear Mixed Model (GLMM) approach will be adopted, using STATA statistical software. Initial analyses will be simple, unadjusted comparisons of individuals. If there appears to be substantial imbalances between individuals in terms of baseline covariates, adjusted analyses will also be performed. All variables that are p<0.15 in the GLMM univariate analyses will be entered into multivariate models using a stepwise approach. All effects will be estimated with 95% confidence intervals, with the threshold for statistical significance determined as a two tailed p-value less than or equal to 0.05. All participants with at least one set of follow-up oral health literacy-related outcome data will be included in multivariate modelling.

### Power calculation

The initial study design utilised a calculation of sample size performed using PC-SIZE software (GE Dallal, 1990, Version 3). Based on the 2008 oral health literacy survey using REALD-30 [10], it was estimated that a sample size of 310 would be necessary to detect a 7.5 percent difference in the proportion of problem-based dental attenders (pre-intervention vs post-intervention), a 25 percent difference in the proportion of those who believe teeth should be brushed none or once daily (pre-intervention vs post-intervention) and a 30 percent difference in the proportion of those who believe cordial is good for teeth, don’t own a toothbrush or own a toothbrush but didn’t brush the previous day (pre-intervention vs post-intervention) at the significance criterion of 0.05 and a power of 0.80. Allowing for an attrition rate of 25 percent after 18 months, 388 participants would be necessary at base-line, rounded up to 400 for convenience; 200 in the intervention group and 200 in the control (delayed intervention) group.

During planning stages of the study, it was evident that some changes were necessary to study design. Specifically, a cluster randomisation approach was more appropriate for this study and community, the HeLM [18] a more appropriate and acceptable tool for assessing oral health literacy than the REALD-30, and a 12-month follow-up more practicable than the proposed 9-month follow-up. The original calculation was retained as the best indicator of sample size available in the absence of other data to perform a revised power calculation.

### Ethics

Ethical approval was granted by the Aboriginal Health Council of South Australia and the Human Research Ethics Committee of the University of Adelaide. The Board of Management of the Pika Wiya Health Service (PWHS), the local community controlled Indigenous health service, also gave approval for the study. Comprised of representatives from the Indigenous community, the Board of Management is the peak body which governs the delivery of PWHS services and programs.

## Discussion

This study will be the first study using a functional, context-specific oral health literacy intervention to improve oral health literacy-related outcomes amongst rural-dwelling Indigenous adults in Australia. Outcomes of this study will have implications for policy and planning by providing evidence for the effectiveness of such interventions as well as providing a model for working with Indigenous communities.

The design of this study has implications for future research where utilising a randomised, controlled study design with Indigenous communities is planned. The delayed intervention study design makes a controlled trial acceptable for the community and increases the proportion of the community potentially benefiting from the intervention.

Consistent with national recommendations for research with Indigenous communities, partnerships between the community and researchers have enabled community feedback to be incorporated into the design of the study. The grouping of participants into clusters was endorsed as the most appropriate method by the advisory group. The questionnaire instruments measuring oral health literacy were changed from REALD-30 to an adapted form of the HeLM in response to feedback from the pilot study as it was deemed to be more acceptable and applicable. It is anticipated that these partnerships will enable greater success of the study and facilitate improved outcomes for the community and future partnerships [24,25]. In addition, the study has sought to involve local Indigenous staff wherever possible. The work of the Indigenous project officers, who are considered community champions, has enhanced the acceptability of the study to the local community, increased the potential for participant involvement and begun to build local research capacity. Substantial efforts have been made to ensure full participation of study participants is supported both through the design of study instruments and ensuring study protocols take into account practical considerations specific to the local community.

## Competing interests

The authors declare no competing interests.

## Authors’ contributions

EJP participated in study design, co-ordinated data collection and data management, and participated in manuscript preparation. GM, AC, KRT and AMH provided important intellectual input into the study design and revision of the manuscript. HM co-ordinated the pilot study, manages the parent project and was involved in manuscript preparation. LJ participated in study design, ethics applications, data management and participated in manuscript preparation. All authors were involved in revising the manuscript for important intellectual content and read and approved the final manuscript.

## Pre-publication history

The pre-publication history for this paper can be accessed here:

http://www.biomedcentral.com/1471-2458/12/461/prepub
